# Supplementary material for: Exact density matrix elements for a driven dissipative system described by a quadratic Hamiltonian
Source: Sci Rep. 2021 Aug 30;11:17388. doi: 10.1038/s41598-021-96787-6 (PMC8405690; doi:10.1038/s41598-021-96787-6)
Supplement: Supplementary file 1 — Supplementary Information. [file 41598_2021_96787_MOESM1_ESM.pdf]

Supplemental file: "Exact density matrix elements for a driven dissipative system described by a quadratic Hamiltonian"

Sh. Saedi[1] and F. Kheirandish*[1]

[1] *Department of Physics, University of Kurdistan, P.O.Box 66177-15175, Sanandaj, Iran*

(Dated: August 8, 2021)

I.

By making use of the Heisenberg equations of motion we have

$$\dot{\hat{a}} = \frac{1}{i\hbar}\left[\hat{a}, \hat{H}\right] = -i\,\omega_0\hat{a} - i\sum_j f_j\hat{b}_j - i\,k(t) - 2\,i\,\phi\hat{a}^\dagger, \tag{1}$$

$$\dot{\hat{b}}_j = \frac{1}{i\hbar}\left[\hat{b}_j, \hat{H}\right] = -i\,\omega_j\hat{b}_j - i\,\bar{f}_j\hat{a}. \tag{2}$$

Eq. (2) can be solved formally as

$$\hat{b}_j(t) = e^{-i\omega_j t}\,\hat{b}_j(0)\, - i\,\bar{f}_j\int_0^t dt'\, e^{-i\omega_j(t-t')}\,\hat{a}(t'), \tag{3}$$

by inserting Eq. (3) into Eq. (1) we will find

$$\dot{\hat{a}} + i\,\omega_0\hat{a} + \int_0^t dt'\,\chi(t-t')\,\hat{a}(t') = -i\sum_j f_j e^{-i\omega_j t}\hat{b}_j(0) - i\,k(t) - 2\,i\,\phi\hat{a}^\dagger(t), \tag{4}$$

where

$$\chi(t-t') = \sum_j |f_j|^2 e^{-i\omega_j(t-t')}, \tag{5}$$

is the response function or the memory function of the medium. By taking the Laplace transform of both sides of Eq. (4) we have

$$\tilde{a}(s) = \left[\frac{1}{s + i\,\omega_0 + \tilde{\chi}(s)}\right]\hat{a}(0) - \left[\frac{i}{s + i\,\omega_0 + \tilde{\chi}(s)}\right]\sum_j f_j\left[\frac{1}{s + i\,\omega_j}\right]\hat{b}_j(0)$$
$$- \left[\frac{2\,i\,\phi}{s + i\,\omega_0 + \tilde{\chi}(s)}\right]\tilde{a}^\dagger(s) - \left[\frac{i}{s + i\,\omega_0 + \tilde{\chi}(s)}\right]\tilde{k}(s), \tag{6}$$

where

$$\tilde{\chi}(s) = \mathcal{L}\Big[\sum_j |f_j|^2 e^{-i\omega_j t}\Big]. \tag{7}$$

Now we can rewrite Eq. (6) and its adjoint as

$$\tilde{a}(s) = \left[\frac{1}{\tilde{G}(s)}\right]\hat{a}(0) - \left[\frac{i}{\tilde{G}(s)}\right]\sum_j\left[\frac{f_j}{s + i\,\omega_j}\right]\hat{b}_j(0) - \left[\frac{2\,i\,\phi}{\tilde{G}(s)}\right]\tilde{a}^\dagger(s) - \left[\frac{i}{\tilde{G}(s)}\right]\tilde{k}(s), \tag{8}$$

$$\tilde{a}^\dagger(s) = \left[\frac{1}{\bar{\tilde{G}}(s)}\right]\hat{a}^\dagger(0) + \left[\frac{i}{\bar{\tilde{G}}(s)}\right]\sum_j\left[\frac{\bar{f}_j}{s - i\,\omega_j}\right]\hat{b}_j^\dagger(0) + \left[\frac{2\,i\,\bar{\phi}}{\bar{\tilde{G}}(s)}\right]\tilde{a}(s) + \left[\frac{i}{\bar{\tilde{G}}(s)}\right]\bar{\tilde{k}}(s), \tag{9}$$

leading to

$$\tilde{a}(s) = \left[\frac{\bar{\tilde{G}}(s)}{\tilde{L}(s)}\right]\hat{a}(0) - \left[\frac{2\,i\,\phi}{\tilde{L}(s)}\right]\hat{a}^\dagger(0) - \left[\frac{i\bar{\tilde{G}}(s)}{\tilde{L}(s)}\right]\sum_j\left[\frac{f_j}{s + i\,\omega_j}\right]\hat{b}_j(0)$$
$$+ \left[\frac{2\,\phi}{\tilde{L}(s)}\right]\sum_j\left[\frac{\bar{f}_j}{s - i\,\omega_j}\right]\hat{b}_j^\dagger(0) - \left[\frac{i\bar{\tilde{G}}(s)}{\tilde{L}(s)}\right]\tilde{k}(s) + \left[\frac{2\,\phi}{\tilde{L}(s)}\right]\bar{\tilde{k}}(s). \tag{10}$$

Now using the inverse Laplace transform we find

$$\hat{a}(t) = \alpha_1(t)\hat{a}(0) - 2\,i\,\phi\alpha_2(t)\hat{a}^\dagger(0) - i\sum_j M_j(t)\hat{b}_j(0) - i\sum_j\left(2\,i\,\phi\right)N_j(t)\hat{b}_j^\dagger(0) - i\,\zeta_1(t) - i\left(2\,i\,\phi\right)\,\zeta_2(t), \tag{11}$$

and from Eq. (3) we deduce

$$\hat{b}_j(t) = \sum_k\left[\Lambda_{jk}(t)\hat{b}_k(0) + \Lambda'_{jk}(t)\hat{b}_k^\dagger(0) - \Gamma_{jk}(t)\hat{a}(0) - \Gamma'_{jk}(t)\hat{a}^\dagger(0) - \Omega_{jk}(t)\right]. \tag{12}$$

II.

We have

$$\hat{Q}_{nm} = \hat{U}^{\dagger}(t)\left(|m\rangle\langle n| \otimes I_R\right)\hat{U}(t), \\ = \hat{U}^{\dagger}(t)\frac{\hat{a}^{\dagger}(0)^m}{\sqrt{m!}}|0\rangle\langle 0|\frac{\hat{a}(0)^n}{\sqrt{n!}} \otimes I_R\hat{U}(t), \tag{13}$$

and [1]

$$|0\rangle\langle 0| = \sum_{s=0}^{\infty}\frac{(-1)^s}{s!}\left(\hat{a}^{\dagger}(0)\right)^s\left(\hat{a}(0)\right)^s, \tag{14}$$

by inserting Eq. (14) into Eq. (13) we obtain

$$\hat{Q}_{nm} = \frac{1}{\sqrt{m!n!}}\sum_{s=0}^{\infty}\frac{(-1)^s}{s!}\hat{U}^{\dagger}(t)\left(\hat{a}^{\dagger}(0)\right)^{m+s}\left(\hat{a}(0)\right)^{n+s} \otimes I_R\,\hat{U}(t), \\ = \frac{1}{\sqrt{m!n!}}\sum_{s=0}^{\infty}\frac{(-1)^s}{s!}\left(\hat{a}^{\dagger}(t)\right)^{m+s}\left(\hat{a}(t)\right)^{n+s}. \tag{15}$$

III.

We have

$$I_{\hat{B}} = \mathrm{Tr}_R\left\{e^{i\lambda\hat{B}^{\dagger}(t)}e^{i\bar{\lambda}\hat{B}(t)}\hat{\rho}_R(0)\right\}, \tag{16}$$

where

$$\hat{B}(t) = \sum_j\left(M_j(t)\hat{b}_j(0) + 2\,i\,\phi\,N_j(t)\hat{b}_j^{\dagger}(0)\right), \\ \hat{B}^{\dagger}(t) = \sum_j\left(\bar{M}_j(t)\hat{b}_j^{\dagger}(0) - 2\,i\,\bar{\phi}\,\bar{N}_j(t)\hat{b}_j(0)\right), \\ \hat{\rho}_R(t) = \frac{1}{Z_R}\prod_j e^{-\beta\hbar\omega_j\hat{b}_j^{\dagger}\hat{b}_j}. \tag{17}$$

By inserting Eqs. (17) into Eq. (16) we obtain

$$I_{\hat{B}} = \prod_k \mathrm{Tr}_k\left\{e^{\left(i\,\lambda\bar{M}_k(t)\hat{b}_k^{\dagger}(0)+2\,\lambda\,\bar{\phi}\,\bar{N}_k(t)\hat{b}_k(0)\right)}e^{\left(i\,\bar{\lambda}M_k(t)\hat{b}_k(0)-2\,\bar{\lambda}\,\phi\,N_k(t)\hat{b}_k^{\dagger}(0)\right)}\frac{e^{-\beta\hbar\omega_k\hat{b}_k^{\dagger}\hat{b}_k}}{Z_k}\right\}, \tag{18}$$

where Tr_k means taking trace over the Hilbert space of the kth oscillator in the environment and Z_k is the corresponding partition function. By using the following equations

$$e^{\mu\hat{b}_k(0)+\nu\hat{b}_k^{\dagger}(0)} = e^{\mu\hat{b}_k(0)}e^{\nu\hat{b}_k^{\dagger}(0)}e^{-\frac{1}{2}\mu\,\nu}, \\ e^{\mu\hat{b}_k(0)}e^{\nu\hat{b}_k^{\dagger}(0)} = e^{\nu\hat{b}_k^{\dagger}(0)}e^{\mu\hat{b}_k(0)}e^{\mu\nu}, \tag{19}$$

we have

$$I_{\hat{B}} = \prod_k\frac{1}{Z_k}\sum_{n_k=0}^{\infty}e^{-\beta\hbar\omega_k n_k}\sum_{m=0}^{n_k}\frac{(-1)^2|\lambda|^{2m}|V_k(t)|^{2m}}{(m!)^2}\frac{n_k!}{(n_k-m)!}e^{i(\lambda^2\,\bar{\phi}\bar{N}_k(t)\bar{M}_k(t)-\bar{\lambda}^2\phi N_k(t)M_k(t))-4|\lambda|^2|\phi|^2|N_k(t)|^2}, \\ = e^{\sum_k\left[-\lambda\,\bar{\lambda}\left(\frac{|V_k(t)|^2}{e^{\beta\hbar\,\omega_k}-1}+4|\phi|^2\,|N_k(t)|^2\right)+i(\lambda^2\,\bar{\phi}\bar{N}_k(t)\bar{M}_k(t)-\bar{\lambda}^2\phi N_k(t)M_k(t))\right]} = e^{\vartheta[\lambda,\bar{\lambda},t]}. \tag{20}$$

IV.

We have

$$\hat{C}(t) = \alpha_1(t)\hat{a}(0) - 2\,i\,\phi\,\alpha_2(t)\hat{a}^\dagger(0), \tag{21}$$

therefore,

$$\begin{aligned} I_{\hat{C}} &= \mathrm{Tr}_S\left\{e^{\lambda\left(\bar{\alpha}_1(t)\hat{a}^\dagger(0)+2\,i\,\bar{\phi}\,\alpha_2(t)\hat{a}(0)\right)}e^{-\bar{\lambda}\left(\alpha_1(t)\hat{a}(0)-2\,i\,\phi\,\alpha_2(t)\hat{a}^\dagger(0)\right)}\,\hat{\rho}_S(0)\right\}, \\ &= \mathrm{Tr}_S\left\{e^{\lambda\,\bar{\alpha}_1(t)\hat{a}^\dagger(0)}e^{2\,i\,\lambda\,\bar{\phi}\alpha_2(t)\hat{a}(0)}e^{i\,\lambda^2\bar{\phi}\,\bar{\alpha}_1(t)\alpha_2(t)}e^{-\bar{\lambda}\alpha_1(t)\hat{a}(0)}e^{2\,i\,\bar{\lambda}\phi\,\alpha_1(t)\hat{a}^\dagger(0)}e^{i\,\bar{\lambda}^2\phi\,\alpha_1(t)\alpha_2(t)}\,\hat{\rho}_S(0)\right\}, \\ &= e^{\left(i\lambda^2\bar{\phi}\,\bar{\alpha}_1(t)-i\,\bar{\lambda}^2\phi\,\alpha_1(t)-4\,\lambda\bar{\lambda}|\phi|^2\alpha_2(t)\right)\alpha_2(t)}\mathrm{Tr}_S\left\{e^{\sigma\hat{a}^\dagger}e^{-\bar{\sigma}\hat{a}}\hat{\rho}_S(0)\right\}, \end{aligned} \tag{22}$$

where

$$\begin{aligned} \sigma &= \lambda\bar{\alpha}_1(t) + 2\,i\,\bar{\lambda}\phi\alpha_2(t), \\ \bar{\sigma} &= \bar{\lambda}\alpha_1(t) - 2\,i\,\lambda\bar{\phi}\bar{\alpha}_2(t). \end{aligned} \tag{23}$$

The normal and anti-normal characteristic functions in quantum optics are respectively defined by

$$\begin{aligned} \mathrm{Tr}_S\left\{e^{\sigma\hat{a}^\dagger}e^{-\bar{\sigma}\hat{a}}\hat{\rho}_S(0)\right\} &= C_N(\sigma), \\ \mathrm{Tr}_S\left\{e^{-\bar{\sigma}\hat{a}}e^{\sigma\hat{a}^\dagger}\hat{\rho}_S(0)\right\} &= C_A(\sigma), \end{aligned} \tag{24}$$

and are related as $C_N(\sigma) = e^{|\sigma|^2}\,C_A(\sigma)$. Let us assume that the initial state of the system is a coherent state

$$\hat{\rho}_S(0) = |\,\gamma\,\rangle\langle\,\gamma\,|, \tag{25}$$

then

$$C_A(\sigma) = \int d^2\alpha\,Q(\alpha)e^{\sigma\,\bar{\alpha}-\bar{\sigma}\,\alpha}, \tag{26}$$

where $Q(\alpha)$ is the Husimi distribution function

$$Q(\alpha) = \frac{\langle\,\alpha\,|\hat{\rho}_S(0)|\,\alpha\,\rangle}{\pi} = \frac{e^{-|\alpha-\gamma|^2}}{\pi}. \tag{27}$$

By inserting Eq. (27) into Eq. (26) we have

$$\begin{aligned} C_A(\sigma) &= \frac{1}{2\pi}\int d^2\alpha\,e^{-|\alpha-\gamma|^2}e^{\sigma\,\bar{\alpha}-\bar{\sigma}\,\alpha} \\ &= \frac{1}{2\pi}\int d^2\alpha\,e^{-|\alpha|^2-|\gamma|^2+(\gamma+\sigma)\bar{\alpha}+(\bar{\gamma}+\bar{\sigma})\alpha}, \\ &= \frac{e^{-|\gamma|^2}}{2\pi}\int d\,q\,e^{-\frac{q^2}{2}+\frac{1}{\sqrt{2}}[(\gamma+\bar{\gamma})+(\sigma-\bar{\sigma})]q}\int d\,p\,e^{-\frac{p^2}{2}+\frac{-i}{\sqrt{2}}[(\gamma-\bar{\gamma})+(\sigma+\bar{\sigma})]p}, \\ &= e^{-|\sigma|^2}e^{\bar{\gamma}\sigma-\gamma\bar{\sigma}}, \end{aligned} \tag{28}$$

therefore,

$$\begin{aligned} \mathrm{Tr}_S\left\{e^{\lambda\hat{C}^\dagger}e^{-\bar{\lambda}\hat{C}}\hat{\rho}_S(0)\right\} =& e^{\left(i\lambda^2\bar{\phi}\,\bar{\alpha}_1(t)+i\,\bar{\lambda}^2\phi\,\alpha_1(t)-4\,\lambda\bar{\lambda}|\phi|^2\alpha_2(t)-2\,i\,\bar{\lambda}^2\phi\alpha_1(t)\right)\alpha_2(t)} \\ &\times e^{\bar{\gamma}\left(\lambda\bar{\alpha}_1(t)+2\,i\,\bar{\lambda}\phi\alpha_2(t)\right)}e^{-\gamma\left(\bar{\lambda}\alpha_1(t)-2\,i\,\lambda\bar{\phi}\alpha_2(t)\right)}. \end{aligned} \tag{29}$$

V.

To calculate I, we define on the complex plane

$$\begin{aligned} \lambda &= u + i\,v, \qquad \bar{\lambda} = u - i\,v, \\ \partial_\lambda &= \frac{1}{2}\partial_u + \frac{1}{2\,i}\partial_v, \qquad \partial_{\bar{\lambda}} = \frac{1}{2}\partial_u - \frac{1}{2\,i}\partial_v, \end{aligned} \tag{30}$$

then

$$\partial_\lambda \partial_{\bar{\lambda}} = \frac{1}{4}\left(\partial_u^2 + \partial_v^2\right) = \frac{1}{4}\nabla^2_{(u,v)}. \tag{31}$$

By definition we have

$$I(u,v) = e^{-\eta(t)\left(u^2+v^2\right)+(u+i\,v)\bar{Z}-(u-i\,v)Z}, \tag{32}$$

using the Fourier transform

$$I(u,v) = \int\int d^2\vec{k}\, e^{(i\,k_u\,u+i\,k_v\,v)}\tilde{I}(k_u,k_v), \tag{33}$$

one finds

$$\begin{aligned} e^{\partial_\lambda\partial_{\bar{\lambda}}} I(\lambda,\bar{\lambda}) &= e^{\frac{1}{4}\nabla^2_{(u,v)}}\, I(u,v), \\ &= \int\int d\,k_u\, d\,k_v\, e^{-\frac{1}{4}\left(k_u^2+k_v^2\right)+i\,k_u\,u+i\,k_v\,v}\tilde{I}(k_u,k_v), \\ &= g(u,v). \end{aligned} \tag{34}$$

Therefore,

$$\begin{aligned} P_n(t)|_{\phi=0} &= \frac{(-1)^n}{n!}\left(\frac{\partial}{\partial\lambda}\frac{\partial}{\partial\bar{\lambda}}\right)^n e^{\partial_\lambda\partial_{\bar{\lambda}}} I(\lambda,\bar{\lambda})\Big|_{\lambda=\bar{\lambda}=0}, \\ &= \frac{1}{(-4)^n n!}(\nabla^2)^n g(u,v)\Big|_{u=v=0}. \end{aligned} \tag{35}$$

Now from the inverse Fourier transform

$$\begin{aligned} \tilde{I}(k_u,k_v) &= \frac{1}{(2\,\pi)^2}\int\int d\,u\,d\,v\, e^{-i(k_u\,u+k_v\,v)} I(u,v), \\ &= \frac{1}{(2\,\pi)^2}\int d\,u\, e^{-\eta(t)u^2} e^{\left((\bar{Z}-Z)-i\,k_u\right)u}\int d\,v\, e^{-\eta(t)v^2} e^{\left(i(\bar{Z}+Z)-i\,k_v\right)v}, \end{aligned} \tag{36}$$

we have

$$\tilde{I}(k_u,k_v) = \frac{1}{4\pi\eta(t)} e^{\left(\frac{-Z\bar{Z}}{\eta(t)}\right)} e^{-\left(\frac{k_u^2}{4\eta(t)}+\frac{i(\bar{Z}-Z)k_u}{2\eta(t)}\right)} e^{-\left(\frac{k_v^2}{4\eta(t)}-\frac{(\bar{Z}+Z)k_v}{2\eta(t)}\right)}, \tag{37}$$

leading to

$$\begin{aligned} g(u,v) &= \left[-\frac{e^{\left(\frac{Z\bar{Z}}{-\eta(t)}\right)}}{4\pi\eta(t)}\int d\,k_u\, e^{\left(\frac{1}{-4\eta(t)}-\frac{1}{4}\right)k_u^2} e^{\frac{i}{-2\eta(t)}\left(\bar{Z}-Z-2\eta(t)\,u\right)k_u}\int d\,k_v\, e^{\left(\frac{1}{-4\eta(t)}-\frac{1}{4}\right)k_v^2} e^{-\frac{1}{2\eta(t)}\left(\bar{Z}+Z+2\,i\,\eta(t)\,v\right)k_v}\right], \\ &= -\frac{1}{-\eta(t)-1} e^{\frac{Z\bar{Z}}{-\eta(t)-1}}\left[e^{-\frac{\eta(t)}{\eta(t)+1}\left(u^2+v^2\right)} e^{-\frac{\bar{Z}-Z}{-\eta(t)-1}u} e^{i\frac{\bar{Z}+Z}{-\eta(t)-1}v}\right]. \end{aligned} \tag{38}$$

Therefore,

$$P_n(t)|_{\phi=0} = \frac{e^{-\frac{|Z|^2}{1+\eta(t)}}}{(-4)^n n!(1+\eta(t))}(\nabla^2_{(u,v)})^n e^{\frac{-\eta(t)u^2-\eta(t)v^2+(\bar{Z}-Z)u\,-\,i(\bar{Z}+Z)v}{1+\eta(t)}}\Big|_{u=v=0}. \tag{39}$$

Now using the following identities

$$\begin{aligned}
&\nabla^2_{(u,v)} = \partial_u^2 + \partial_u^2,\\
&[\partial_u, \partial_v] = 0,\\
&\left(\partial_u^2 + \partial_u^2\right)^n e^{\frac{-\eta(t)u^2+\left(\bar{Z}-Z\right)u}{1+\eta(t)}} e^{\frac{-\eta(t)v^2-i\left(\bar{Z}+Z\right)v}{1+\eta(t)}} = \sum_{k=0}^{\infty} \binom{n}{k} \left(\partial_u^2\right)^{n-k} e^{\frac{-\eta(t)u^2+\left(\bar{Z}-Z\right)u}{1+\eta(t)}} \left(\partial_v^2\right)^k e^{\frac{-\eta(t)v^2-i\left(\bar{Z}+Z\right)v}{1+\eta(t)}},\\
&e^{-l^2+2\,x\,l} = \sum_{n=0}^{\infty} H_n(x)\frac{l^n}{n!},\\
&\partial_l^n \left(e^{-l^2+2\,x\,l}\right)|_{l=0} = H_n(x),
\end{aligned} \tag{40}$$

and the definitions

$$\begin{aligned}
&-\frac{\eta(t)u^2}{\eta(t)+1} = -l^2, \qquad u = \sqrt{\frac{\eta(t)+1}{\eta(t)}}l,\\
&x = \frac{\bar{Z}-Z}{2\sqrt{\eta(t)(\eta(t)+1)}},\\
&-\frac{\eta(t)v^2}{\eta(t)+1} = -l'^2, \qquad v = \sqrt{\frac{\eta(t)+1}{\eta(t)}}l',\\
&x' = -i\frac{Z+\bar{Z}}{2\sqrt{\eta(t)(\eta(t)+1)}},
\end{aligned} \tag{41}$$

we will find

$$\begin{aligned}
e^{\left(\frac{-\eta(t)u^2+(\bar{Z}-Z)u}{1+\eta(t)}\right)} &= \sum_{p=0}^{\infty} H_p\Big(\frac{\bar{Z}-Z}{2\sqrt{\eta(t)(\eta(t)+1)}}\Big)\frac{\left(\sqrt{\frac{\eta(t)}{\eta(t)+1}}\,u\right)^p}{p!},\\
e^{\left(\frac{\eta(t)v^2-i(\bar{Z}+Z)v}{1-\eta(t)}\right)} &= \sum_{q=0}^{\infty} H_q\Big(\frac{-i(Z+\bar{Z})}{2\sqrt{\eta(t)(\eta(t)+1)}}\Big)\frac{\left(\sqrt{\frac{\eta(t)}{\eta(t)+1}}\,v\right)^q}{q!},
\end{aligned} \tag{42}$$

so

$$\begin{aligned}
&\nabla^{2\,n}\left(e^{\frac{-\eta(t)u^2+\left(\bar{Z}-Z\right)u}{1+\eta(t)}} e^{\frac{-\eta(t)v^2-i\left(\bar{Z}+Z\right)v}{1+\eta(t)}}\right)\Big|_{u=v=0} =\\
&\sum_{k=0}^{n}\binom{n}{k}\left[\partial_u^{2(n-k)}\sum_{p=0}^{\infty}H_p\left(\frac{\bar{Z}-Z}{2\sqrt{\eta(t)(\eta(t)+1)}}\right)\left(\sqrt{\frac{\eta(t)}{\eta(t)+1}}\right)^p\frac{u^p}{p!}\right]_{u=0}\\
&\qquad\times\left[\partial_v^{2\,k}\sum_{q=0}^{\infty}H_q\left(\frac{-i(Z+\bar{Z})}{2\sqrt{\eta(t)(\eta(t)+1)}}\right)\left(\sqrt{\frac{\eta(t)}{\eta(t)+1}}\right)^q\frac{v^q}{q!}\right]_{v=0},\\
&=\sum_{k=0}^{n}\binom{n}{k}\left(\frac{\eta(t)}{\eta(t)+1}\right)^n H_{2\,n-2\,k}\left(\frac{\bar{Z}-Z}{2\sqrt{\eta(t)(\eta(t)+1)}}\right)H_{2\,k}\left(\frac{-i(Z+\bar{Z})}{2\sqrt{\eta(t)(\eta(t)+1)}}\right).
\end{aligned} \tag{43}$$

Finally, using the identity

$$\sum_{k=0}^{n}\binom{n}{k}H_{2\,n-2\,k}(x)H_{2\,k}(y) = (-4)^n n!\,L_n(x^2+y^2), \tag{44}$$

we deduce that

$$
\begin{aligned}
P_n(t)|_{\phi=0} &= \frac{e^{-\frac{|Z|^2}{1+\eta(t)}}}{(-4)^n n!(1+\eta(t))}\left(\frac{\eta(t)}{\eta(t)+1}\right)^n \left[(-4)^n n!\, L_n\left(\frac{(Z-\bar{Z})^2-(Z+\bar{Z})^2}{4\eta(t)(\eta(t)+1)}\right)\right] \\
&= \frac{e^{-\frac{|Z|^2}{1+\eta(t)}}}{(1+\eta(t))}\left(\frac{\eta(t)}{\eta(t)+1}\right)^n L_n\left(\frac{-|Z|^2}{\eta(t)(1+\eta(t))}\right), \qquad (45)
\end{aligned}
$$

where $L_n[x]$ is a Laguerre polynomial of degree n.

---

[1] W. H. Louisell, Quantum Statistical Properties of Radiation (Wiley, New York, 1975)
